# Oculomotor nerve palsy caused by imidacloprid at initial diagnosis: A case report

**DOI:** 10.1097/MD.0000000000039160

**Published:** 2024-08-02

**Authors:** Huan Jiang, Xiaoli Bu, Taixiang Liu, Bing Jiang

**Affiliations:** a Department of Ophthalmology, Affiliated Hospital of Zunyi Medical University, Zunyi City, China; b Department of Critical Care Medicine, Affiliated Hospital of Zunyi Medical University, Zunyi City, China.

**Keywords:** imidacloprid, oculomotor nerve palsy, poisoning, ptosis

## Abstract

**Rationale:**

Amid the pervasive deployment of imidacloprid, the incidence of poisoning from this compound has risen markedly. Those afflicted with imidacloprid poisoning typically exhibit symptoms ranging from headaches, dizziness, nausea, and abdominal pain, to impaired consciousness and breathlessness, yet instances of ocular paralysis induced by this toxin have not previously been documented.

**Patient concerns:**

When the pesticide spray inadvertently made contact with the patient’s eyes, they were seared with a burning sensation and discomfort. Subsequent to this incident, on the second day, the individual began to experience diplopia in the right eye and found it arduous to elevate his eyelids, indicating a challenge in achieving full extension.

**Diagnoses:**

Based on the medical history, symptoms, and signs, the patient was diagnosed with oculomotor nerve palsy caused by imidacloprid.

**Interventions:**

The treatment involved intravenous dexamethasone to reduce inflammatory response in the eye tissue; oral pantoprazole enteric-coated tablets to suppress acid production and protect the stomach; Xuesaitong administered intravenously to improve blood supply to the eye and promote metabolism of toxins; vitamin C, cobamamide, and vitamin B1 for nerve nutrition and antioxidant effects; local application of tobramycin-dexamethasone eye drops for anti-inflammatory purposes; and repeated flushing of the conjunctival sac with saline. Finally, the patient improved and was discharged.

**Outcomes:**

After active treatment, the patient finally improved diplopia and ptosis.

**Lessons:**

This report marks the first documentation of oculomotor nerve palsy induced by imidacloprid, featuring diplopia, and blepharoptosis without substantial limitation of ocular motility. Following therapeutic intervention, the patient showed marked improvement and was discharged from the hospital, providing a point of reference for the treatment of analogous cases in future clinical practice. It also serves as a reminder for the public to take appropriate precautions when using imidacloprid.

## 1. Introduction

Imidacloprid, a neonicotinoid insecticide, exerts its toxicity by selectively targeting nicotinic acetylcholine receptors (nAChRs) within insects. Its mechanism of action involves binding to these receptors, which in turn leads to an accumulation of acetylcholine within the insect’s body, ultimately causing paralysis and subsequent death due to overstimulation of the nervous system.^[1]^ These nAChRs are widely expressed throughout both the central and peripheral nervous systems, serving as prime targets for imidacloprid’s effects. In humans, exposure to imidacloprid can result in symptoms resembling nicotine intoxication, with severe instances potentially leading to neurological damage.^[2]^ Moreover, the liver and kidneys, being pivotal metabolic organs, incur varying degrees of damage from imidacloprid’s toxic effects. This damage manifests as typical hepatic cell degeneration, capillary hyperemia, and edema, alongside harm to renal tubular epithelial cells and necrotic changes.^[3,4]^

The clinical presentations of imidacloprid poisoning are highly varied, with mild cases presenting with symptoms such as fatigue, nausea, vomiting, drowsiness, dizziness, and headache, while severe cases may involve life-threatening complications including hemorrhage, cardiac arrest, gastrointestinal hemorrhage, respiratory failure, liver and kidney failure, coma, and even death.^[5,6]^ A review of clinical studies on imidacloprid poisoning from around the world indicates a strong correlation between the occurrence of aspiration pneumonia, respiratory failure, liver and kidney failure, neurotoxic effects, and cardiac dysfunction with the duration of hospitalization, the probability of intensive care unit admission, and mortality rates.^[7–9]^ Notwithstanding the lack of documented cases of ocular paralysis due to imidacloprid poisoning in the literature, this article provides a detailed account of a case of ocular paralysis resulting from imidacloprid exposure, discussing the diagnostic approach, therapeutic interventions, and the underlying mechanisms, with the aim of offering a reference for the diagnosis and treatment of related conditions that may follow.

## 2. Case report

A 65-year-old male patient was hospitalized due to diplopia and the inability to fully open his right eye, which had persisted for 4 days. Five days prior to symptom onset, he had been applying a 1- to 2-thousand-fold dilution of imidacloprid pesticide to fruit trees. Accidentally, the pesticide entered his eye during application, causing immediate burning and discomfort, yet he did not seek medical attention at that time. Subsequently, on the second day postexposure, he developed diplopia and found it challenging to open his right eye, experiencing occasional itchiness without a sensation of foreign bodies or pain, along with slightly limited eye movement. His left eye remained asymptomatic. The patient reported no symptoms of cough, sputum production, dizziness, headache, or limb weakness. Following the onset, he was treated at a local clinic without improvement, prompting admission to our facility. The patient denied any history of diabetes or hypertension.

**Figure 1.**
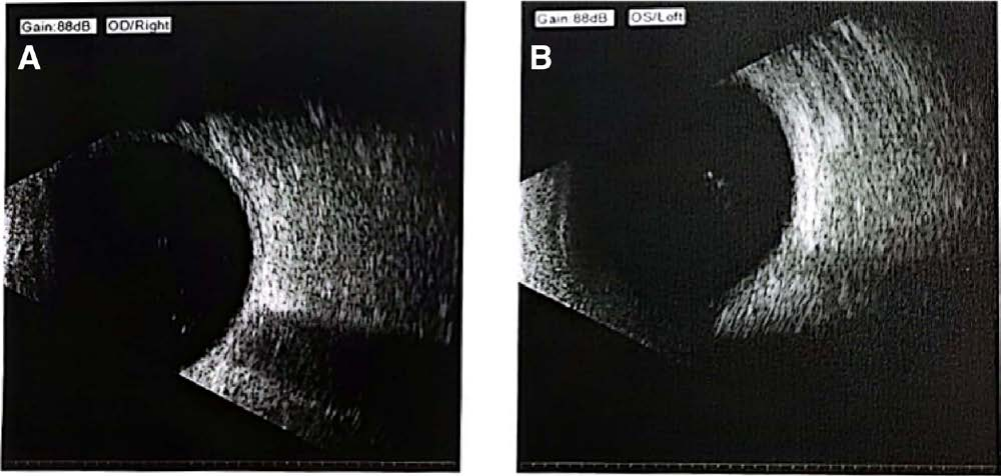
B-ultrasound of both eyes on admission day. (A) Right eye. (B) Left eye.

**Figure 2.**
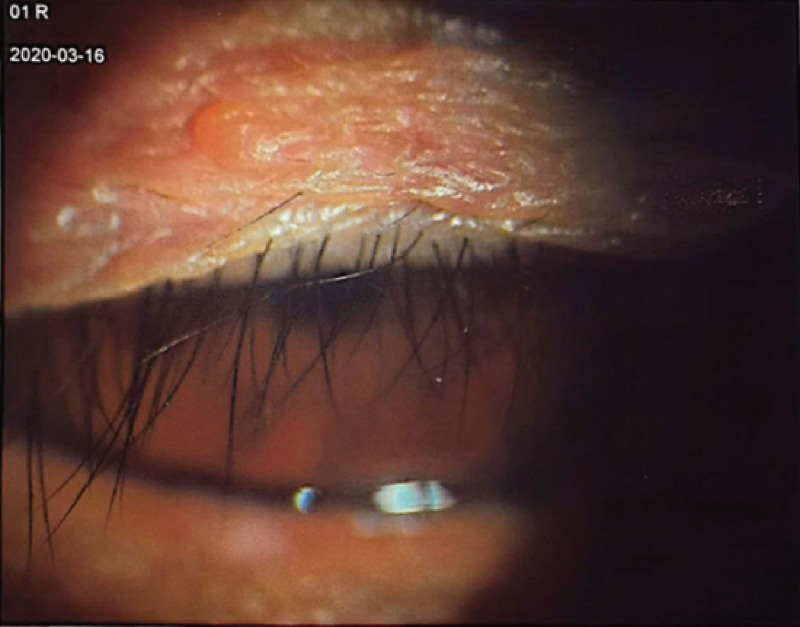
Anterior segment photograph taken with the patient forcibly opening the right eyes.

On physical examination, no significant abnormalities were detected. B-mode ultrasonography of both eyes indicated vitreous opacities, with no other remarkable findings (Fig. 1). Ophthalmological evaluation revealed visual acuities of 1.0 in each eye. The right upper eyelid partially obscured the cornea (Fig. 2). Notably, upward and rightward gazes were restricted in the right eye, accompanied by conjunctival congestion, a clear cornea, a round pupil measuring approximately 3 mm, diminished direct and consensual light reflexes, an intact lens, and an unremarkable fundus. His left eye appeared normal. The admitting diagnosis was right ocular motor paralysis.

**Figure 3.**
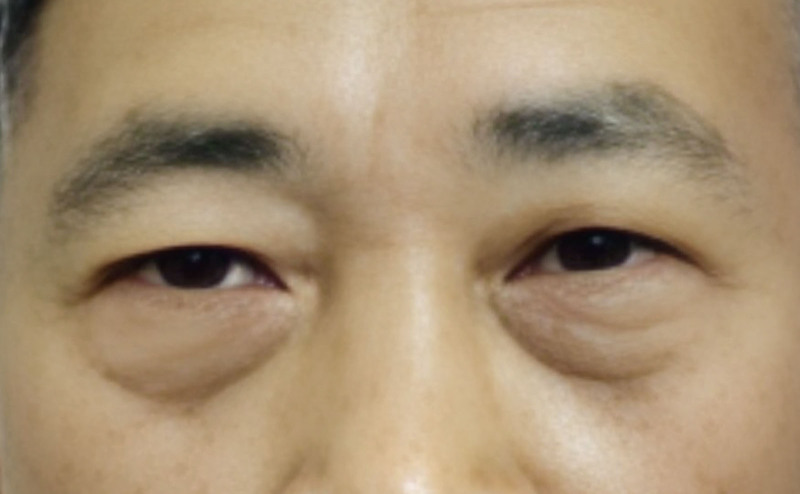
The patient’s right eye returned to normal after treatment.

On the day of admission, routine examinations such as chest computed tomography (CT), head CT, electrocardiogram, complete blood count, and liver and kidney function tests were conducted and no obvious abnormalities were found. On the first day of treatment, dexamethasone 5 mg was administered intravenously once daily to reduce inflammatory response in the eye; pantoprazole enteric-coated tablets 40 mg were given orally once daily to protect the stomach; Xuesaitong 400 mg was administered intravenously once daily to improve blood supply to the eye and promote metabolism of toxic substances; vitamin C 2 g was injected intravenously once daily to nourish nerves; tobramycin-dexamethasone eye drops were used locally to anti-inflammatory; and the conjunctival sac was flushed repeatedly with saline. On the second day, the patient’s diplopia and ptosis showed no improvement, so the dexamethasone dose was adjusted to 10 mg intravenously once daily, with the same treatment as before. On the third day, upon reviewing the patient’s diplopia and ptosis had not significantly improved, the treatment plan was adjusted to dexamethasone 20 mg injected intravenously multiple times to flush the conjunctival sac with saline, oral cobamamide 0.5 mg (3 times/d), and vitamin B1 0.1 g injected intramuscularly (twice/day) to nourish nerves, with the same treatment as before. On the fourth day, the patient’s diplopia was slightly relieved and ptosis slightly improved, with the same treatment as before. On the fifth day, the patient reported no diplopia and significant improvement in ptosis, so the dexamethasone dose was adjusted to 5 mg, and the patient was discharged on the seventh day with instructions to continue using tobramycin-dexamethasone eye drops at home. On the 13th day, a phone call follow-up revealed that the size of the right eyelid aperture was the same as that of the left eye (Fig. 3). The specific treatment measures and effects are detailed in Table 1.

**Table 1:**
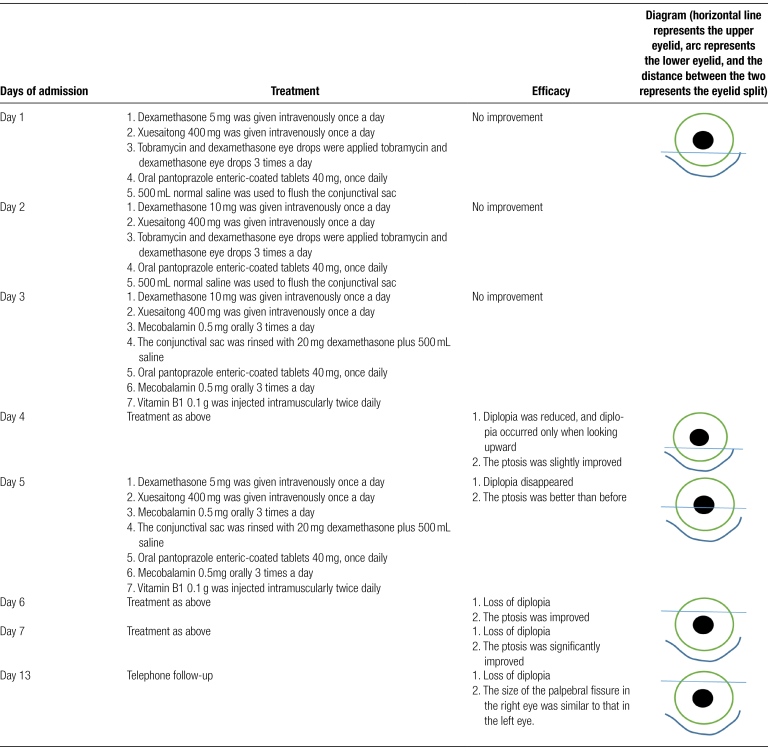
Treatment process and effects.

## 3. Discussion

Pyraclostrobin is a novel neonicotinoid insecticide known for its low toxicity and high efficiency, particularly effective against insects that have developed resistance to organophosphate and carbamate pesticides. Its primary mode of action involves the selective inhibition of nAChRs in insect nervous systems, leading to paralysis, and eventual death.^[10]^ However, with its extensive use in agricultural settings, incidents of human and animal poisoning have become more frequent. Recent studies have highlighted the serious health risks associated with pyraclostrobin poisoning, which, despite being relatively rare, can lead to severe health issues. For instance, a report documented 4 cases of pyraclostrobin poisoning admitted to a tertiary care center, with 2 resulting in multiple organ failure and death, demonstrating the severe toxicity of the pesticide.^[11]^ Another study showed that pyraclostrobin poisoning may cause liver damage, with a possible dose-response relationship in the severity and type of hepatic injury, noting that initial liver tests were normal but delayed liver damage could occur.^[12]^ A separate study focused on pyraclostrobin poisoning cases referred to the Mahidol Poison Center in Bangkok, Thailand, between 2010 and 2018. It was found that most exposures were through ingestion, presenting primarily with gastrointestinal symptoms, while neurological impacts were less common. Most outcomes were mild or nontoxic, yet some cases exhibited cholinergic syndrome-like symptoms, liver damage, and even death, with a mortality rate of 3.1%.^[8]^ This suggests that even mild exposures can have fatal consequences. Current literature predominantly reports cases of pyraclostrobin poisoning via gastrointestinal exposure, with treatments typically including gastric lavage, the administration of atropine to reduce glandular secretions, and blood purification techniques to clear the body of pyraclostrobin, thereby increasing the survival rate of severely poisoned patients.

To date, there have been no reported cases of oculomotor nerve paralysis caused by pyraclostrobin poisoning. In this case, the patient developed oculomotor nerve palsy after local exposure of the eyes to pyraclostrobin, presenting primarily with diplopia and ptosis, with no other special discomfort. The treatment involved intravenous dexamethasone to reduce inflammatory response in the eye tissue; oral pantoprazole enteric-coated tablets to suppress acid production and protect the stomach; Xuesaitong administered intravenously to improve blood supply to the eye and promote metabolism of toxins; vitamin C, cobamamide, and vitamin B1 for nerve nutrition and antioxidant effects; local application of tobramycin-dexamethasone eye drops for anti-inflammatory purposes; and repeated flushing of the conjunctival sac with saline. Ultimately, the patient recovered and was discharged from the hospital.

Most cases of pyraclostrobin poisoning result from exposure via the digestive tract, yet instances of oculomotor nerve palsy are uncommon. This is mainly due to the presence of the blood-aqueous barrier and blood-retinal barrier, which collectively function as the blood-eye barrier, preventing drugs circulating in the peripheral blood from reaching effective concentrations within the ocular tissues. Additionally, when patients inadvertently consume pyraclostrobin, they usually receive immediate gastric lavage upon hospitalization, with blood purification therapy administered when necessary to avert further increases in drug concentration in the eye, thus mitigating the risk of damaging the oculomotor nerve. In this particular case, the patient sustained direct ocular damage from pyraclostrobin, manifesting swiftly with symptoms of acute conjunctivitis. After a certain latent period, the individual developed right eyelid ptosis and diplopia, the latter characterized as mixed type. The affected eye’s vision remained unaffected. The patient had no recent history of catching a cold or flu and no diabetic background. Upon admission, routine examinations revealed no significant abnormalities. Coupled with the patient’s definite history of pyraclostrobin exposure, a definitive diagnosis of oculomotor nerve palsy attributable to pyraclostrobin poisoning was established.

Recent research suggests that oculomotor nerve palsy caused by pyraclostrobin may share similar mechanisms with botulinum toxin-induced muscle paralysis, where the toxin absorbs through the conjunctiva and enters the gap between the Muller muscle and the levator palpebrae superioris synapses, inhibiting the release of acetylcholine from the presynaptic membrane of motor nerve endings, causing flaccid paralysis of the muscles.^[13]^ Alternatively, the toxin may act on acetylcholine receptors, preventing acetylcholine from binding to acetylcholine receptors, leading to paralysis of the muscles innervated by the oculomotor nerve. Regarding the neurotoxicity of pyraclostrobin, researchers have found that it can induce neurotoxicity in rats by blocking acetylcholinesterase activity and reducing dopamine, serotonin, and γ-aminobutyric acid levels.^[14]^ Furthermore, pyraclostrobin elevates malondialdehyde levels and impairs antioxidant capacity, upregulates the transcription levels of nuclear factor-κB, interleukin-1β, and tumor necrosis factor genes, and expresses high caspase-3 and inducible nitric oxide synthase. This study also shows that essential oil from Origanum vulgare and its extracts can counteract the neurotoxicity of pyraclostrobin, preventing its neurotoxic effects through their potent antioxidant, anti-inflammatory, and antiapoptotic properties.^[14]^ A research team employed untargeted lipidomics and metabolomics techniques to uncover a new mechanism by which pyraclostrobin and another neonicotinoid insecticide, dinotefuran, exert toxic effects on Neuro-2a neural cells. These insecticides disrupt various lipid and amino acid metabolisms within cells, leading to cellular dysfunction and affecting the body’s normal antioxidant and immune regulatory functions, thereby inducing various diseases and adverse reactions, including neural weakness.^[15]^ Additionally, research has found that chronic low-dose exposure to pyraclostrobin leads to anxiety and depression-like behaviors in zebrafish, characterized by significantly reduced exploration of new environments, increased decision-making time, and decreased social activities. Disruption of the circadian rhythm in zebrafish leads to metabolic imbalances and neurotransmitter disturbances, which are potential mechanisms of pyraclostrobin’s neurobehavioral toxicity.^[16]^ These studies provide new insights into the neurotoxicity mechanisms of pyraclostrobin and offer scientific bases for assessing its toxicological risks and developing preventive measures.

The oculomotor nerve, also known as the third cranial nerve, is a motor nerve that contains both somatic and visceral efferent fibers. It originates from the ventral interpeduncular fossa of the midbrain, runs close to the edge of the tentorium cerebelli and the lateral aspect of the posterior clinoid process, passes through the upper part of the cavernous sinus, and enters the orbit through the superior orbital fissure, controlling the movement of the extraocular muscles.^[17]^ Any damage along its course can result in oculomotor nerve palsy. When differentiating the clinical presentation of this patient, we should consider the following conditions: Myasthenia gravis: an autoimmune disease that commonly affects women aged 20 to 40, presenting initially with ptosis and diplopia, but without the typical pattern of worsening in the evening and improvement in the morning. Given the clear history of pyraclostrobin exposure and the absence of the characteristic fluctuation, this condition can be differentiated.

Diabetic oculomotor nerve palsy: usually the result of a combination of factors, primarily microvascular complications of diabetes leading to ischemia and hypoxia. However, the patient’s acute onset, lack of diabetes history, and normal blood sugar levels upon admission make this diagnosis unlikely. Viral infection: although viral infections can cause oculomotor nerve palsy, the patient has a clear history of pyraclostrobin intoxication and no recent history of a cold or flu, so this possibility is not considered at this time.

Limitations of this case report include the slight restriction of the patient’s eye movement upward, while full range of motion was observed in other directions. However, we did not capture supportive photographs of the patient’s gaze.

Through the reporting of this case, we have gained an understanding that local exposure to pyraclostrobin can result in injury to the oculomotor nerve, manifesting symptoms such as ptosis and diplopia. Following the aforementioned active treatments, the patient’s diplopia and ptosis showed significant improvement, offering valuable references for the diagnosis and management of similar cases in the future. This case also serves as a reminder for individuals in agricultural settings to take appropriate precautions to prevent direct exposure to pyraclostrobin, thereby reducing related hazards.

## 4. Conclusion

Imidacloprid has been identified to potentially cause oculomotor nerve palsy, manifesting primarily as diplopia and ptosis. With aggressive treatment, significant recovery can be achieved.

## Acknowledgments

The authors sincerely thank the family for giving permission to report this case.

## Author contributions

**Writing—original draft:** Huan Jiang.

**Conceptualization:** Xiaoli Bu.

**Writing—review & editing:** Taixiang Liu, Bing Jiang.

## References

[1] Campbell KS Keller PG Heinzel LM. Detection of imidacloprid and metabolites in Northern Leopard frog (Rana pipiens) brains. Sci Total Environ. 2022;813:152424. 34942261 10.1016/j.scitotenv.2021.152424

[2] Forrester MB. Neonicotinoid insecticide exposures reported to six poison centers in Texas. Hum Exp Toxicol. 2014;33:568–73. 24513674 10.1177/0960327114522500

[3] Zheng M Qin Q Zhou W. Metabolic disturbance in hippocampus and liver of mice: a primary response to imidacloprid exposure. Sci Rep. 2020;10:5713. 32235887 10.1038/s41598-020-62739-9 PMC7109098

[4] Arfat Y Mahmood N Tahir MU. Effect of imidacloprid on hepatotoxicity and nephrotoxicity in male albino mice. Toxicol Rep. 2014;1:554–61. 28962268 10.1016/j.toxrep.2014.08.004 PMC5598541

[5] Lin PC Lin HJ Liao YY Guo H-R Chen K-T. Acute poisoning with neonicotinoid insecticides: a case report and literature review. Basic Clin Pharmacol Toxicol. 2013;112:282–6. 23078648 10.1111/bcpt.12027

[6] Yeh IJ Lin TJ Hwang DY. Acute multiple organ failure with imidacloprid and alcohol ingestion. Am J Emerg Med. 2010;28:255.e1–3. 10.1016/j.ajem.2009.05.006 20159407

[7] Mohamed F Gawarammana I Robertson TA. Acute human self-poisoning with imidacloprid compound: a neonicotinoid insecticide. PLoS One. 2009;4:e5127. 19352499 10.1371/journal.pone.0005127 PMC2662424

[8] Sriapha C Trakulsrichai S Tongpoo A Pradoo A Rittilert P Wananukul W. Acute imidacloprid poisoning in Thailand. Ther Clin Risk Manag. 2020;16:1081–8. 33204096 10.2147/TCRM.S269161 PMC7667159

[9] Perananthan V Mohamed F Shahmy S Gawarammana I Dawson A Buckley N. The clinical toxicity of imidacloprid self-poisoning following the introduction of newer formulations. Clin Toxicol (Phila). 2021;59:347–50. 32959700 10.1080/15563650.2020.1815760

[10] Taillebois E Thany SH. The use of insecticide mixtures containing neonicotinoids as a strategy to limit insect pests: efficiency and mode of action. Pestic Biochem Physiol. 2022;184:105126. 35715064 10.1016/j.pestbp.2022.105126

[11] Naveen A Sahu MR Padhi KS Maharik SS. Self-poisoning with safer insecticide: a case series on imidacloprid poisoning. Am J Forensic Med Pathol. 2022;43:66–8. 33950883 10.1097/PAF.0000000000000685

[12] Sriapha C Trakulsrichai S Intaraprasong P. Imidacloprid poisoning case series: potential for liver injury. Clin Toxicol (Phila). 2020;58:136–8. 31092066 10.1080/15563650.2019.1616091

[13] Tang S Hu Y Wang Y Lu J Yang B. One patient of blepharoptosis caused by levator palpebrae superioris aponeurosis degeneration. J Craniofac Surg. 2022;33:e866–9. 35864575 10.1097/SCS.0000000000008799

[14] Hassanen EI Issa MY Hassan NH. Potential mechanisms of imidacloprid-induced neurotoxicity in adult rats with attempts on protection using *Origanum majorana* L. oil/extract: in vivo and in silico studies. ACS Omega. 2023;8:18491–508. 37273614 10.1021/acsomega.2c08295 PMC10233680

[15] Wang X Qiu J Xu Y. Integrated non-targeted lipidomics and metabolomics analyses for fluctuations of neonicotinoids imidacloprid and acetamiprid on Neuro-2a cells. Environ Pollut. 2021;284:117327. 34030083 10.1016/j.envpol.2021.117327

[16] Liu H Fu R Zhang Y. Integrate transcriptomic and metabolomic analysis reveals the underlying mechanisms of behavioral disorders in zebrafish (Danio rerio) induced by imidacloprid. Sci Total Environ. 2023;870:161541. 36731560 10.1016/j.scitotenv.2023.161541

[17] Park HK Rha HK Lee KJ Chough CK Joo W. Microsurgical anatomy of the oculomotor nerve. Clin Anat. 2017;30:21–31. 27859787 10.1002/ca.22811

